# Genetic Diversity of Transcription Factor Genes in *Triticum* and Mining for Promising Haplotypes for Beneficial Agronomic Traits

**DOI:** 10.3389/fpls.2022.899292

**Published:** 2022-07-08

**Authors:** Guang Yang, Ying Zhang, Xinyu Wei, Licao Cui, Xiaojun Nie

**Affiliations:** ^1^State Key Laboratory of Crop Stress Biology in Arid Areas, College of Agronomy, Northwest A&F University, Yangling, China; ^2^College of Biological Science and Engineering, Jiangxi Agricultural University, Nanchang, China

**Keywords:** genetic diversity, haplotype, transcription factor, wheat, yield traits

## Abstract

Transcription factor (TF) is a class of the sequence-specific DNA-binding proteins that modulate the transcription of target genes, and thus regulate their expressions. Variations in TF are the crucial determinants for phenotypic traits. Although much progress has been made in the functions of TF genes in wheat, one of the most important staple crops globally, the diversity of TF genes in wheat and its progenitors are not well understood, especially the agronomically promising haplotypes have not yet been characterized. Here, we identified a total of 6,023 TF genes from hexaploid wheat through a genome-search method and classified them into 59 gene families based on the conserved domain. The characteristics and dN/dS values of these genes showed evidently selective effects. Based on re-sequencing data, we found a strong genetic bottleneck among these TF genes on A and D subgenomes while no found in B subgenome during wheat domestication. Combined with selective signals and known QTLs on the whole genome, 21 TF genes were preliminarily found to be associated with yield-related traits. The haplotype frequency of these TF genes was further investigated in bread wheat and its progenitors and 13 major haplotypes were the casual loci related to key traits. Finally, the tissue-specific TF genes were also identified using RNA-seq analysis. This study provided insights into the diversity and evolution of TF genes and the identified TF genes and excellent haplotypes associating with traits will contribute to wheat genetic improvement.

## Introduction

Bread wheat (*Triticum aestivum* L., AABBDD) is one of the most important staple crops in the world, representing 20% of the total protein and calories consumption in human (Sansaloni et al., 2020). With global population growth and climate change, improvement of wheat yield has been a key focus of breeding programs over the past several decades (Camargo et al., 2016; Dong et al., 2019; Liu et al., 2020a), and an ambitious 60% increase in wheat production by 2050 has been targeted to meet the predicted demand for wheat (FAO) (reviewed in Tucker et al., 2017). Successful identification of major genes and quantitative trait loci (QTLs) underlying agronomic traits is a prerequisite for improving wheat yield. During the last decade, many QTLs and genes for yield-related traits in wheat have been isolated using genome sequence and variation data. For example, genes associated with enlarging sink size have been identified by evaluating yield-related traits such as grain size, spikelet number, and plant height (Zhai et al., 2018; Kuzay et al., 2022; Xu et al., 2022).

Variation in gene expression is a main driver of phenotypic evolution (Tirosh et al., 2009). Quantitative traits are influenced by gene dosage variation in nature (Bastiaanse et al., 2021). For instance, an indel variation (540 bp in length) in the promotor region of *ahb2* was significantly associated with gene expression and the expression of *ahb2* was negatively associated with drought tolerance in 224 maize accessions (Liu et al., 2020b). However, in addition to the differences in expression caused by genetic variation of functional genes themselves, variation in gene expression between germplasms can also be caused by variation in transcriptional regulatory factor (Nallu et al., 2014). Sequence-specific transcription factor (TF) that can bind to a specific promoter motif upstream of the transcription initiation site is a key component of RNA polymerase II and controls the transcription of protein coding genes (Mittler et al., 2009; Downs et al., 2013). In plants, TF genes are important components of regulatory cascades in responses to development stress (Singh et al., 2002; Wu et al., 2021). Some transcription factors act as hubs of transcription networks, and trigger multiple biochemical and developmental pathways in wheat. For instance, a 108-bp insertion contains two MYB (myeloblastosis) cis-regulatory elements present in the promoter of *TaNAC071-A*, a member of NAC (NAM/ATAF/CUC) transcription factor family. These two cis-regulatory elements that can be bound directly by *TaMYBL1* for transcriptional activation of *TaNAC071-A*, which confers drought tolerance (Mao et al., 2022). *TaWRKY51*, a member of the plant-specific WRKY transcription factor family, plays a key role in lateral root formation by modulating ethylene biosynthesis and is a positive regulator of traits contributing to grain yield (Hu et al., 2018; Li et al., 2021). Moreover, domestication and improvement have affected variation in the expression of key trait-related genes. In a Maize-Teosinte population, seven *Bx* genes for benzoxazinoid biosynthesis showed consistent regulatory divergence between maize and teosinte. Of these, maize alleles of six *Bx* genes were consistently associated with higher benzoxazinoid biosynthesis than were teosinte alleles, and one *Bx* gene with teosinte alleles was associated with higher benzoxazinoid biosynthesis expression than the maize allele, suggesting that the *Bx* genes underwent regulatory divergence during maize domestication or improvement (Wang et al., 2018). Thus, it is also important to investigate the diversity and variation of key genes during evolution. Although recent advances in genomic data and population variation data collection have yielded new prospects for understanding plant function and evolution, few studies have investigated the diversity and haplotypes of trait-related TF genes in wheat domestication and improvement.

In this study, we identified 59 TF gene families in common wheat and investigated differences in basic characteristic and dN/dS ratios between TF and non-TF genes. The diversity and major haplotype of TF genes throughout domestication and improvement were further captured using whole-genome sequencing data, and selective pressure, known QTLs, and transcriptome data were integrated in order to identify promising functional TF genes and key haplotypes for several agronomic traits. This study lays a foundation for investigations of wheat TF gene diversity at the genus level in *Triticum* and provides important haplotype resources of trait-related TF genes for future wheat breeding and improvement.

## Materials and Methods

### Identification of TF Genes in Wheat

All protein sequences (FASTA file) and information about genomic positions (GFF file) in the hexaploid wheat reference genome “Chinese Spring” (IWGSC RefSeq v1.1) were downloaded from EnsemblPlants.^1^ To get the TF genes of wheat, a total of 56 wheat TF gene families were collected from the Plant Transcription Factor Database (PlantTFDB). However, due to the incomplete annotation in PlantTFDB, some TF genes have been annotated as the low confidence genes in IWGSC RefSeq v1.1. Therefore, after filtering the low confidence TF genes, 53 families were retained. Moreover, six plant-specific TF gene families, namely Alfin-like, OFP, PLATZ, HB-BELL, LIM, and Tify, were further searched. Furthermore, we obtained the seed alignment file for 59 domains from the PFAM database^2^ (El-Gebali et al., 2019) and built HMM files based on the seed file using HMMER (version 3.3.2)^3^ (Potter et al., 2018). Then, we used the HMMsearch function of HMMER to search the annotated protein database of the reference genome and retained all possible results (E-value ≤ 1e-5). A total of 6,023 sequences from 59 TF gene families were identified. The domain information of all genes was further confirmed using SMART^4^ (Letunic et al., 2021), InterPro^5^ (Blum et al., 2021), EnsemblPlants database and iTAK program (Zheng et al., 2016). Of these, splice isoforms were excluded and only the first variant was retained for further analysis. We evaluated the predicted gene structure of all genes using in-house Python scripts and the physicochemical properties using the ExPASy server^6^ (Artimo et al., 2012). Homoelogous genes among A, B and D subgenomes were identified using OrthoFinder (version 2.5.4) (Emms and Kelly, 2019) with default parameters.

### Orthologous and Molecular Selection Analysis

BLASTP was used to identify orthologs (from *Triticum urartu*, *Triticum dicoccoides*, and *Aegilops tauschii*) with the reciprocal best hits method with an E-value ≤ 1e-5 (Altschul et al., 1997), and the gene pairs with the highest degree of sequence similarity were retained (Yang et al., 2011). Furthermore, CDSs and protein sequences of orthologous TF gene pairs were submitted to ParaAT2.0 software to obtained the input files for PAML program. Non-synonymous rates (dN), synonymous rates (dS), and the ratio of non-synonymous to synonymous substitution rates (dN/dS) were estimated using the codeml module of PAML (version 4.9e) (Yang, 2007) with the following parameters: F3 × 4 codon frequency model; codon model = 2; runmode = −2; seqtype = 1; CodonFreq = 2; model = 2; cleandata = 1. We restricted our data set to orthologous gene pairs with dS < 0.3 (Yang et al., 2011). The formula [T = (dS/2λ) × 10^–6^) Mya] was used to calculate the divergence time, where λ referred to the mutation rate and was considered as 6.5 × 10^–9^ synonymous substitutions per site per year.

### Genetic Diversity and Selective Signals in *Triticum* and *Aegilops*

Nucleotide variation data from 261 *Triticum* and *Aegilops* accessions, including wild einkorn (*Triticum monococcum*, 31), domesticated einkorn (31), urartu (*T. urartu*, 29), wild emmer (*Triticum dicoccoides*, 28), domesticated emmer (*Triticum dicoccon*, 29), durum wheat (*Triticum durum*, 13), landrace (*T. aestivum*, 45), cultivar (*T. aestivum*, 25), and *Ae. tauschii* (*Aegilops tauschii* Coss. ssp. *strangulate*, 9; *Aegilops tauschii* Coss. ssp. *tauschii* var. *meyeri*, 11; *Aegilops tauschii* Coss. ssp. *tauschii* var. *anathera*, 10), were extracted from 414 *Triticum* accessions (Zhou et al., 2020). SNPs located within TF genes were extracted based on the chromosome location using an in-house Python script. Nucleotide diversity (π) and fixation index (*F*st) were calculated using VCFtools (version 0.1.16) (Danecek et al., 2011) with a sliding window of 50 Kb. The windows with the top 5% of π ratios (domestication process: π_wild_/π_domesticated_; improvement process: π_domesticated_/π_cultivar_) and top 5% of *F*st values were considered as candidate selective region.

### Haplotype Analysis of TF Genes

Nucleotide variations of TF genes were extracted from the VCF files. SNPs located within the upstream, downstream, CDS, and UTR regions were included. Genotype imputation was phased using BEAGLE (version 5.3) software (Browning et al., 2018). To identify the major haplotype of each TF gene, we constructed the haplotype of TF genes in nine subgroups (wild einkorn; domesticated einkorn; urartu; wild emmer; domesticated emmer; durum; landrace; cultivar; *Ae. tauschii*) using in-house Python scripts. Haplotypes with > 50% of the total number of subgroups were considered as the major haplotypes.

### Function Analysis

The previously reported QTLs associated with 14 wheat agronomic traits (grain yield, GY; grain color, GC; kernel number per spike, KNS; kernel weight, KW; kernel length, KL; spikelet number per spike, SNS; reaction to *Puccinia graminis* Pers, SR; sprouting, SP; leaf spot disease, LSD; reaction to leaf rust, LR; reaction to *Puccinia striiformis* Westend, YR; plant height, HT; heading date, HD; normalized water index, NWI) were obtained from Cheng et al. (2019). The TF genes were considered potentially related to the agronomic trait if it was located in the QTL associated with that trait. PO (Plant ontology) and TO (Trait ontology) analyses were performed using the clusterProfiler package (Yu et al., 2012). The background terms of PO and TO were download from the Planteome website^7^ (Jaiswal et al., 2005).

### Analysis of RNA-Seq Data

RNA-seq data were download from NCBI database under BioProject accession number: PRJNA497810, PRJNA532455, and PRJNA525250. RNA-seq data from 80 samples were aligned to the “Chinese Spring” genome (IWGSC Refseq v1.1) using HISAT2 (version 2.2.1) (Kim et al., 2015) with default parameters. Transcripts were assembled using StringTie (version 2.1.4) (Pertea et al., 2015). GTF files of each sample were merged using the StringTie-merge tool and the expression levels of the transcripts were evaluated. Transcripts with normalized FPKM > 0 in more than one sample were considered expressed. Co-expression network was analyzed using the WGCNA package (Langfelder and Horvath, 2008). The modules were divided using the following parameters: deepSplit—2, minModuleSize—100, and mergeCutHeight—0.25.

### Tissue Specific Expression Analysis of TF Genes

Expression data were obtained from the NCBI database, which contains samples from 80 different tissues and developmental time points. The TF genes that were expressed (FPKM > 0) in more than one sample were considered expressed. The tissue specificity of all expressed TF genes was measured with the index τ (Yanai et al., 2005):


$$\tau =\frac{\sum_{j=1}^{n}\left[1-l\,o\,g_{2}\,S\,\left(i,j\right)/l\,o\,g\,S_{2}\,\left(i,m\,a\,x\right)\right]}{n-1},$$

where *n* = 80 represents the number of tissue and time points samples and S(i,max) represents the highest expression of gene i across the 80 samples. The index τ ranges from 0 to 1, with higher values indicating higher specificity (or, synonymously, higher variation in expression across libraries). If a gene is expressed in only one library, τ approaches 1. In contrast, if a gene is expressed equally in all libraries, τ = 0 (reviewed in Yang and Gaut, 2011; Yanai et al., 2005).

## Results

### Characteristics of TF Genes in Wheat

**FIGURE 1:**
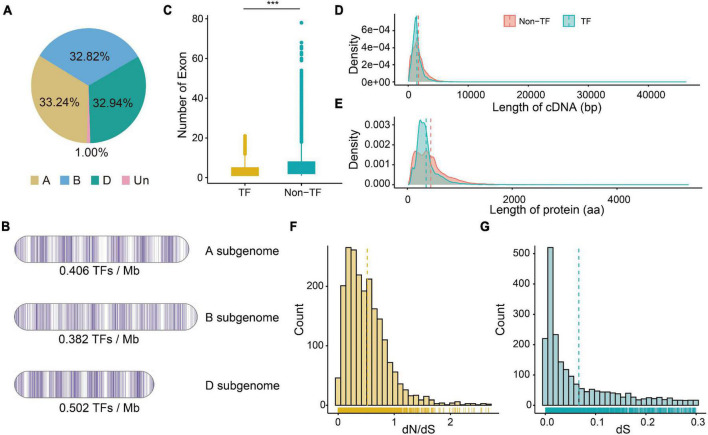
The characteristics of TF genes and the dN (number of substitutions per non-synonymous site) and dS (number of substitutions per synonymous site) values of pairs. **(A)** Percentage of TF genes on three subgenomes. **(B)** Density of TF genes on three subgenomes. **(C)** Numbers of exon of TF and non-TF genes. ***P-value of Student’s test < 0.01. **(D)** Distribution of cDNA length of TF and non-TF genes. **(E)** Distribution of protein length of TF and non-TF genes. **(F)** Distribution of dN/dS ratio of paired TF genes. **(G)** Distribution of dS value of paired TF genes.

A total of 59 gene families consisting of 6,023 TF genes were identified based on functional annotation (PFAM domains) (Supplementary Table 1), and 2,002 (33.24%), 1,977 (32.82%), and 1,984 (32.94%) TFs were identified in the A, B, and D subgenomes, respectively (Figure 1A and Supplementary Table 2). The A, B, and D subgenomes had an average of 0.406, 0.382, and 0.502 TFs per Mb, respectively (Figure 1B). TF genes were most enriched on chromosome 5A (5.22% of the total genes on that chromosome), and were rarely found on chromosome 6D (221 TF genes, 3.67% of the total genes on that chromosome). Among 59 gene families, the bHLH family had the most members, followed by AP2/ERF-ERF (APETALA2/Ethylene-responsive factor), C2H2 (Cys2/His2-type zinc finger proteins), NAC, B3, and MYB, whereas HRT (hypersensitive response of *Turnip crinkle virus*) family possessed fewest members.

Since transcription factors regulates the transcription of downstream gene, both TF and non-TF genes were examined for primary gene characteristics. The TF transcripts were shorter on average (median 2,031 bp; mean 2,918.6 bp) than were the non-TF transcripts (median 2,867 bp; mean 3,995.0 bp) (Supplementary Table 2). The TF transcripts also contained fewer exons than did the non-TF transcripts (Figure 1C), and most of the TF transcripts (96.0%) had less than 10 exons. The cDNA and amino acids regulating TF genes (1,460.9 bp and 357.2 aa) were also shorter than those regulating non-TF genes (1,706.3 bp and 446.6 aa) (Figures 1D,E). The 5′ UTR length of TF genes did not differ from that of non-TF genes, but the 3′ UTR of TF genes was significantly shorter than that of non-TF genes (Supplementary Figures 1A,B). There was no significant difference in the number of isoforms between TF (mean 1.25) and non-TF genes (mean 1.24).

We then identified the A, B, and D homoeologous pairs in these wheat TF family. A total of 1,446 orthologous groups (Supplementary Table 2) with 872 1:1:1 high-confidence homoeologous TF triads were identified. Moreover, the N:N:N (at least one is not 1) homoeologous pairs indicated the complex duplication and loss events among the three subgenomes during the process of wheat polyploidization. We also calculated dN and dS values between wheat and its progenitors. The distribution of dN/dS values showed a significant peak at 0.18 –0.27 (Figure 1F and Supplementary Table 3), which suggested that TF genes have undergone purifying selection. The distribution of dS values showed a significant peak at 0.01 – 0.02 (Figure 1G), approximately 0.77–1.54 MYA. We found that the dN/dS values of TF genes without homoeologous genes (1:0:0 or 0:1:0 or 0:0:1 on A, B, and D subgenomes) were significantly larger than those of triads (1:1:1), suggesting that triads underwent stronger purifying selection compared to the TF genes without homoeologous genes (Supplementary Figure 1C).

### Diversity of TF Genes in the Evolution of *Triticum* and *Aegilops*

**FIGURE 2:**
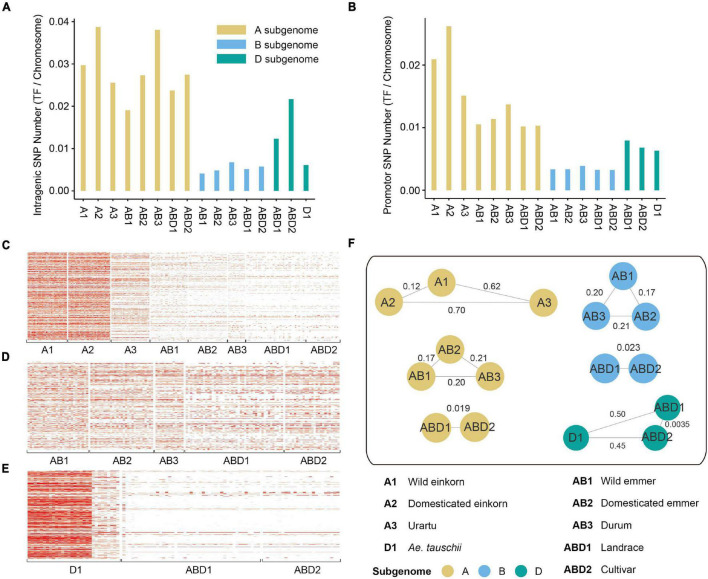
SNP density and population analysis of TF genes. **(A)**. The number of SNPs within TF gene region in each group. **(B)** The number of SNPs within TF promotor region in each group. **(C–E)** The density of SNPs within gene and promotor region on panels **(C)** A, **(D)** B, and **(E)** D subgenomes. **(F)** The *F*st values between different groups. The values on the lines represent *F*st.

To investigate variation in the diversity of TF genes from different *Triticum* and *Aegilops* lineages, we calculated the percentage of SNPs within TF gene and promotor regions (Supplementary Table 4). In total, 4,898 SNPs were located in the gene region and 4,872 SNPs were located in the promotor region, and the number of genic SNPs in the A subgenome was larger than those in the B and D subgenomes. The number of SNPs in the TF gene region was greater than that in the promotor region in all *Triticum* lineages. However, the number of SNPs in the promotor region was greater than that in the TF gene region (Figures 2A,B) in the diploid DD lineages, indicating that TF gene’s expression in *Aegilops* may have a higher degree of variation.

To further evaluate the effect of purifying selection, we calculated the nucleotide diversity (π) and population divergence (*F*st) of each TF gene family based on genotyping of *Triticum* and *Aegilops*. Overall, the A and D subgenomes had stronger bottleneck effects during domestication and improvement (Figures 2C–E). The genetic diversity of TF genes (measured by π) decreased dramatically from wild to domesticated einkorn (2.20 × 10^–4^ for the A subgenome), and from wild to domesticated emmer (0.59 × 10^–4^ for the A subgenome, 1.20 × 10^–4^ for the B subgenome), to durum (0.57 × 10^–4^ for the A subgenome, 1.94 × 10^–4^ for the B subgenome), and to hexaploid landraces (8.32 × 10^–6^ for the B subgenome) (Supplementary Table 5). However, the diversity of hexaploid landraces on the A subgenome was similar to that of wild emmer. It might be explained by the hybridization with its wild relatives during modern breeding processes. The A subgenome harbored higher genetic diversity (0.00022) than the B (0.00012) and D (8.15E-05) subgenomes, and more genetic diversity was lost from *Ae. tauschii* to landrace and cultivar (7.98 × 10^–4^ for the D genome between *Ae. tauschii* and hexaploid landrace). We also observed a severe population divergence of TF genes (measured by *F*st) between wild and cultivated species (Figure 2F and Supplementary Table 5). The *F*st values associated with domestication (0.303) were on average larger than those associated with improvement (0.194). The largest divergence was between domesticated einkorn and urartu (*F*st = 0.70).

### Selective Signals and Major Haplotypes of TF Genes During Domestication and Improvement

To further study signals of selection of TF genes during domestication (wild einkorn versus domesticated einkorn, wild emmer versus domesticated emmer, *Ae. tauschii* versus hexaploid landrace) and improvement (domesticated einkorn versus urartu, domesticated emmer versus durum, hexaploid landrace versus cultivar), we scanned the whole genome for TF gene regions with increased differences in genetic diversity (π ratio) and population divergence to detect candidate selective sweeps. Totally, a 102.40 Mb domesticated-related selective region (A: 71.40 Mb; B: 29.75 Mb; D: 1.25 Mb) and a 76.60 Mb improvement-related selective region (A: 63.70 Mb; B: 12.30 Mb; D: 0.60 Mb) were identified. Fifty-three TF genes were located in the selective region of domestication and 24 TF genes were located in the selective region of improvement. Six of these TF genes were shared by the two processes (Supplementary Table 6). The group of wild versus domesticated emmer had the most TF genes (26, 36.6%), followed by wild versus domesticated einkorn (18, 25.4%) and domesticated emmer versus durum (14, 19.7%).

In order to investigate the relative contributions of the progenitor populations partitioned above to genetic pools of TF genes in bread wheat, we characterized the haplotype of TF genes in bread wheat using genotyping data. In total, 5,280 TF genes were used to construct the haplotype (1,822 in the A, 1,542 in the B, and 1,916 in the D subgenomes). Specifically, 5,031 TF genes (83.5%) had major haplotypes in at least one population (Supplementary Table 7). Among them, 2,560 TF genes (42.50%) (1,552 in the A, 673 in the B, and 335 in the D subgenomes) had different major haplotypes in different populations. For instance, TraesCS1A02G276000, a NAC gene family member, had four haplotypes (CCAACCAA, 82.76%; TTGGCCAA, 10.34%; TTGGTTAA, 3.45%, TCGATCAA, 3.45%) in urartu, and CCAACCAA was the major haplotype. However, after polyploidization, CCAACCAA was occupied by TTGGTTAA in wild emmer (96.43%), domesticated emmer (100%), and durum (100%), and this haplotype was retained after the second polyploidization (hexaploid landrace, 84.44%; hexaploid cultivar, 80.0%). Similarly, TraesCS3B02G404400, a bZIP gene family member, had three haplotypes in wild emmer (CCGG, 75.0%; CCAA, 21.43%; CCAG, 3.57%). However, the major haplotype CCGG was replaced by CCAA in domesticated emmer (89.66%), durum (92.31%), hexaploid landrace (97.78%), and hexaploid cultivar (100%). These results showed that wheat TF genes were subjected to selection pressure during evolution, and some haplotypes were selected for during domestication and improvement.

### Analysis of the Function and Expression of TF Genes

**FIGURE 3:**
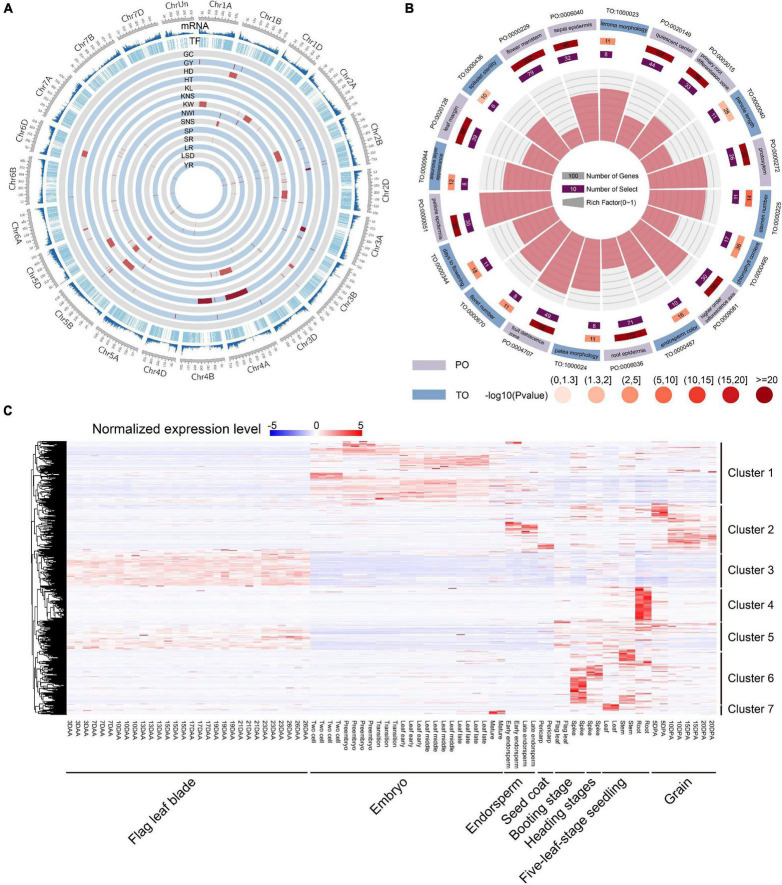
Function analysis and expression heatmap of TF genes. **(A)** Summary of total gene density, TF density, and the distribution of 124 known QTLs of 14 agronomic traits of wheat. **(B)** Top 20 PO and TO enrichment terms of TF genes. **(C)** Expression profiles of TF genes in eight different tissues and time points. Clusters 1–7 on the right represent classification according to gene expression specificity. DAA represents days after anthesis; DPA represents days post-anthesis.

TF genes can indirectly affect wheat traits by regulating the expression of downstream functional genes. To investigate the potential function of TF genes, 124 known QTLs of 14 agronomic traits, including grain yield (GY), grain color (GC), kernel number per spike (KNS), kernel weight (KW), kernel length (KL), spikelet number per spike (SNS), reaction to *Puccinia graminis* Pers (SR), sprouting (SP), leaf spot disease (LSD), reaction to leaf rust (LR), reaction to *Puccinia striiformis* Westend (YR), plant height (HT), heading date (HD), and normalized water index (NWI) were used for co-location analysis (Cheng et al., 2019). A total of 1,148 TF genes were identified to be overlapped with QTLs, and KW had the greatest number of associated genes (Figure 3A and Supplementary Table 8). These results suggested that TF genes were involved in important biological processes and affected the agronomic traits of wheat. By integrating the selective signals, we observed 21 TF genes that were shared by selective region and QTL interval (Supplementary Table 9), indicating that these genes were closely related to important agronomic traits in wheat evolution and were under strong selection pressure. Ten TF genes had different major haplotypes in different populations. Interestingly, seven of these 10 TF genes were located near each other on chromosome 4A, and all were located in the QTL related to KW. Plant ontology (PO) and trait ontology (TO) enrichment analyses for all TF genes showed that many terms related to yield traits were significantly enriched, including days to flowering (TO:0000344, FDR = 3.79E-04), spikelet sterility (TO:0000436, FDR = 0.015), panicle length (TO:0000040, FDR = 0.023), and stamen primordium (PO:0004705, FDR = 6.05E-15) (Figure 3B). These results indicated that the TF gene and its major haplotypes can have important effects on phenotype during both domestication and improvement.

We then investigated the spatial-temporal expression patterns of TF genes using 80 RNA-seq samples from different tissues and stages. A total of 5,767 TF genes were found to be expressed in at least one sample, and some genes exhibited tissue-specific expression. We further used RNA-seq data to estimate TF gene expression levels and tissue specificity. The median tissue specificity index (τ) was 0.84 and mean was 0.77, indicating that most TF genes showed tissue-specific expression profiles (Supplementary Table 1 and Supplementary Figure 1D) and high spatial-temporal expression specificity that depend on its specific function. All of the expressed TF genes were clustered into seven groups (Figure 3C). Of these, TF genes in cluster 1 were primarily expressed in the embryo stage. TF genes in cluster 2 were primarily expressed in endosperm and grain in the post-anthesis stage. TF genes in clusters 3 and 5 were primarily expressed in flag leaf blade in the anthesis stage, but TF genes in cluster 5 in the vegetative and reproductive stages were more expressed than were TF genes in cluster 3. TF genes in clusters 4, 6, and 7 were specifically expressed in the vegetative and reproductive stages, but TF genes in cluster 6 were primarily expressed in the spike and stem, while TF genes in clusters 4 and 7 were expressed in the root and leaf, respectively. Furthermore, a hypergeometric test showed that 40, 22, 28, 16, 21, 30, and 13 TF gene families were significantly enriched in clusters 1-7, respectively (Supplementary Table 2). Furthermore, we constructed the co-expression network for 5,767 expressed TF genes using 80 RNA-seq samples, of which 27 co-expression modules were identified (Supplementary Figure 2). Then, the top 20 downstream co-expressed gene of 4,779 TF genes were screened based on intramodular connectivity, which may be the key potential targets (Supplementary Table 10).

**FIGURE 4:**
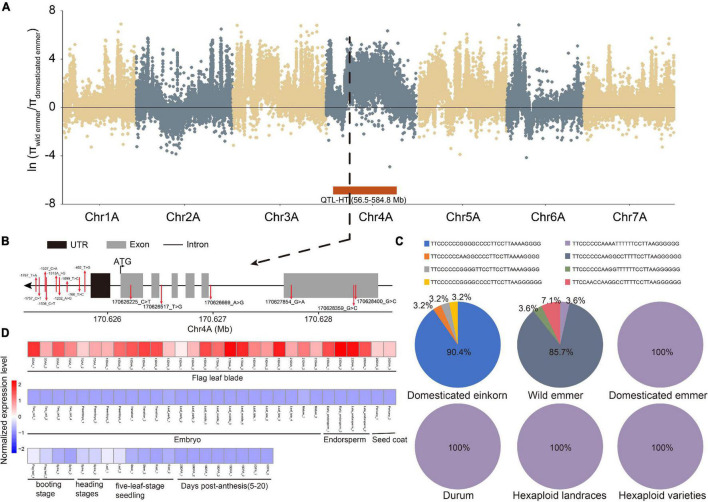
An example of key TF gene TraesCS4A02G130000. **(A)** π ratio between wild and domesticated emmer on the A subgenome. Black dotted line represents the position of TraesCS4A02G130000. Red box represents the position of one QTL related to plant height (HT). **(B)** Gene structure and SNP position of TraesCS4A02G130000. **(C)** Haplotype frequency of TraesCS4A02G130000. **(D)** Heatmap of TraesCS4A02G130000 expression in different tissues and at different developmental time points. DAA represents days after anthesis; DPA represents days post-anthesis.

Finally, we integrated the selective signals, major haplotypes, QTL and expression levels. Thirteen genes overlapped in these analyses, highlighting their importance in wheat evolution and character formation. For example, TraesCS4A02G130000, a member of the GARP-G2-like gene family, was found in the selection region for domestication (wild versus domesticated emmer) and a QTL related to plant height (Figure 4A). Meanwhile, 15 SNPs were identified in TraesCS4A02G130000, and the frequency of the major haplotype (TTCCCCCCAAAATTTTTTCCTTAAGGGGGG) increased during domestication (Figures 4B,C). Based on transcriptome data, this gene was further determined to be specifically expressed in flag leaf blade (Figure 4D), suggesting that it affects plant height. For example, TraesCS5B02G265300, a bZIP gene, was found in the selection region for improvement (hexaploid landrace versus cultivar) and has the major haplotype in durum, hexaploid landrace, and cultivar populations (Supplementary Table 11). At the same time, this gene was found in the QTL related to KW, and the gene expression specificity and module-trait correlation analyses showed that the gene was specifically expressed in the grain.

## Discussion

Transcription factors regulate gene expression by binding specifically to certain DNA sequences. Genome-wide identification and characterization TFs is a crucial step in deciphering transcription regulatory networks. Computational approaches have become the major process to tackle this problem. In this study, we identified 6,023 TF genes from the bread wheat genome and comprehensively investigated the genetic diversity, evolution, and promising haplotype of TF genes using multiple-omics data.

### The Structure of Wheat TF Genes Is Significantly Different From That of Non-TF Genes

Compared with non-TF genes, there were significant differences in transcript, cDNA, protein, 3′UTR length, and exon number of TF genes. In particular, the 3′UTR of TF genes was significantly shorter than that of non-TF genes, but there was no difference in 5′UTR length, suggesting the conservation of 5′UTR length between non-TF and TF genes (Lin and Li, 2012). In contrast, the difference in 3′UTR length showed differences in mRNA stability, translational efficiency, and mRNA localization, as well as protein complex formation (Mayr, 2016; Griesemer et al., 2021), which may depend on the specific regulation functions of the TF genes. Consistent with previous studies (Lin et al., 2010; Dossa et al., 2016; Zhao et al., 2016), the dN/dS values of most TF genes were < 1, indicating that these proteins were undergoing purifying selection during evolution. It is worth noting that dN/dS ratio of triads was significantly lower than other TF genes without homologous genes between subgenomes. We speculated that triads were subjected to longer selection pressure thus have more selection traces on the sequence.

### Genetic Diversity and Bottleneck of TF Genes in *Triticum* and *Aegilops*

Based on genotyping data from 261 *Triticum* and *Aegilops* accessions (Zhou et al., 2020), we obtained the SNPs within gene and promotor regions. The B subgenome was more diverse than were the A and D subgenomes (Cheng et al., 2019; Zhou et al., 2020). However, we found that the diversity of TF SNPs in the A subgenome was greater than that of the B and D subgenomes. The large difference in SNP density was found in the A and D subgenomes during domestication, especially for *Ae. tauschi vs.* hexaploidy landrace and domesticated einkorn *vs.* urartu. Variation in gene promoters correlates with variation in gene expression (Tirosh et al., 2009; Vinces et al., 2009), but mutations in the exons of genes correlate with the function of proteins. Except for in *Ae. tauschii*, more SNPs were found within TF genes than within promotor regions, likely as a result of the many changes in the conserved domain of the TF genes during domestication, which may indirectly affect its function. On the contrary, more SNPs within promotor region suggested that the TF genes in *Ae. tauschii* may have more variation in expression than other populations. These results provided important guidance for researching evolutionary model of TF genes. Moreover, we also found a rapid loss of genetic diversity in TF genes on D subgenome. This was probably due to the severe bottlenecks associated with the domestication and polyploidization of *T. aestivum*.

### Selection Signals and Important Haplotypes Within Wheat TF Genes

Due to the confounding presence of multiple paralogous loci, genome-wide genetic diversity and association analysis based on SNPs had experienced difficulties (Tinker et al., 2016). Nevertheless, the newly updated haplotype-based genetic procedure can deal with the problems effectively. Haplotype-based genetic analyses have been used in human, animal, and plant genetics research (Bekele et al., 2018). In order to reveal the signature of selection pressure due to TF gene variation, we constructed the haplotype for each TF gene using the genic SNPs. There were 5,031 TF genes with a major haplotype in at least one subpopulation, indicating that some haplotypes of these genes are likely to be affected by selection. The haplotype diversity of wild subpopulations was higher than that of cultivated populations, consistent with a previous study in rice (He et al., 2021). Crop domestication is a complex evolutionary process driven by strong artificial selection (reviewed in Zhou et al., 2021), and wheat epitomizes the effectiveness of artificial selection and improvement in shaping a crop to meet human demands and economic incentives (Gegas et al., 2010). Thus, the increased frequency of some haplotypes during domestication and improvement suggests that these haplotypes were selected for and played important roles to humans, especially in yield-related traits. The co-location analysis of known QTLs also showed the importance of these haplotypes. For example, the frequency of haplotype TTCCCCCCAAAATTTTTTCCTTAAGGGGGG within TraesCS4A02G130000 (encoding a GARP-G2-like protein) increased during domestication (wild versus domesticated emmer) and is also occupied the major haplotype in durum and hexaploid wheat, indicating that this haplotype was a potential genetic resource associated with wheat plant height and was retained. In sum, these haplotype resources can be developed into Kasper markers to quickly screen excellent plants in a wider population and accelerate breeding in the future.

### The Potential Function and Downstream Target Genes of Wheat TF Genes

Transcriptome data from different tissues and developmental time points enabled us to get a preliminary insight into the potential functions of TF genes in wheat. Similar to a previous study (Schmitz et al., 2016), most of TF genes showed the specific expression trend in different tissues and development stages, indicating the functional differentiation of different TF families and sub-families. Through integrating multiple-omics data, we found that thirteen TF genes had major haplotypes and were located in the overlapped region of the QTLs and selective regions. Meanwhile, tissue specific expression further identified the potential function of TF genes, such as TraesCS5B02G265300, a member of the bZIP gene family with a major haplotype in cultivated species, which is specifically expressed in wheat grain and is located in the QTL related to kernel weight. As an orthologous gene of TraesCS5B02G265300, *TGA10* has been reported to be involved in the regulation of early and middle tapetal development through the activation of SPOROCYTELESS/NOZZLE gene expression (Murmu et al., 2010). Finally, we also constructed the co-expressed modules and screened the downstream target genes of TF genes. For instance, *ABA1* gene, which was involved in the xanthophyll cycle and abscisic acid (ABA) biosynthesis, has been reported to be targeted by MYB transcription factor in *Arabidopsis*, pineapple and *Populus trichocarpa* (Ding et al., 2009; Chen et al., 2020; Fang et al., 2020). In our study, *ABA1* was co-expressed with MYB genes in our results, which could be selected as an excellent candidate for further molecular breeding in wheat.

## Conclusion

Here, we provide the landscape of diversity and haplotype of TF genes in wheat and its progenitors. We found that TF genes possessed high genetic diversity in wheat wild progenitors, and TF genes had undergone a severe genetic bottleneck during wheat evolution process. Furthermore, population selection effects and known QTL analysis suggested that some TF genes related to important agronomic traits were selected during domestication and improvement. Moreover, we constructed the haplotype of each TF gene and some major haplotypes were located in key selective regions and QTLs. We also profiled the expression pattern using transcriptome data from different tissue and time points and further characterized the functions of some TF genes and their major haplotypes. Our study provided some insights into the evolution of TF gene diversity and also identified the promising haplotypes that may be beneficial to future breeding in wheat.

## Data Availability Statement

The original contributions presented in this study are included in the article/Supplementary Material, further inquiries can be directed to the corresponding authors.

## Author Contributions

XN and GY designed and supervised the project. GY collected and generated the data and perform analysis. YZ helped to prepare the figures and tables. XW performed the WGCNA analysis. GY and LC prepared the draft manuscript. XN reviewed and revised the manuscript. All authors contributed to the article and approved the submitted version.

## Conflict of Interest

The authors declare that the research was conducted in the absence of any commercial or financial relationships that could be construed as a potential conflict of interest.

## Publisher’s Note

All claims expressed in this article are solely those of the authors and do not necessarily represent those of their affiliated organizations, or those of the publisher, the editors and the reviewers. Any product that may be evaluated in this article, or claim that may be made by its manufacturer, is not guaranteed or endorsed by the publisher.

## Footnotes

1
http://plants.ensembl.org/info/data/ftp/index.html

2
http://pfam.xfam.org

3
http://www.hmmer.org

4
https://smart.embl-heidelberg.de/

5
http://www.ebi.ac.uk/interpro/search/sequence/

6
https://web.expasy.org/compute_pi/

7
https://planteome.org/

## Supplementary Material

Supplementary Figure 1 **(A)** Length of 3′ UTR of TF and non-TF genes. **(B)** Length of 5’ UTR of TF and non-TF genes. Student’s test was used to test for statistical significance. **(C)** The dN/dS ration of non-triads and triads. Student’s test was used to test for statistical significance. **(D)** The distribution of tissue specificity indices of TF genes expressed in 80 RNA-seq samples.

Supplementary Figure 2 **(A)** Network analysis dendrogram based on hierarchical clustering of genes by their topological overlap, identifies 27 modules. Colored bars below the dendrogram show module membership. **(B)** Heatmap of correlation between 27 co-expression modules.
