# Supplementary figures and images for: Genetic Diversity of Transcription Factor Genes in Triticum and Mining for Promising Haplotypes for Beneficial Agronomic Traits
Source: Front Plant Sci. 2022 Jul 8;13:899292. doi: 10.3389/fpls.2022.899292 (PMC9305608; doi:10.3389/fpls.2022.899292)

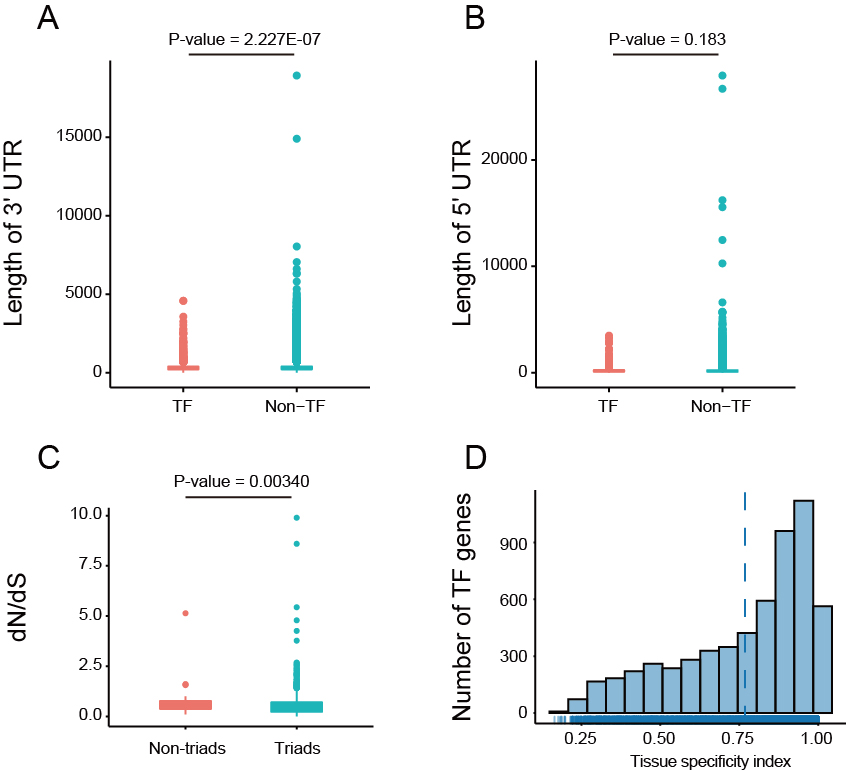

Supplement: Supplementary Figure 1 — (A) Length of 3′ UTR of TF and non-TF genes. (B) Length of 5’ UTR of TF and non-TF genes. Student’s test was used to test for statistical significance. (C) The dN/dS ration of non-triads and triads. Student’s test was used to test for statistical significance. (D) The distribution of tissue specificity indices of TF genes expressed in 80 RNA-seq samples. [file Image_1.JPEG]

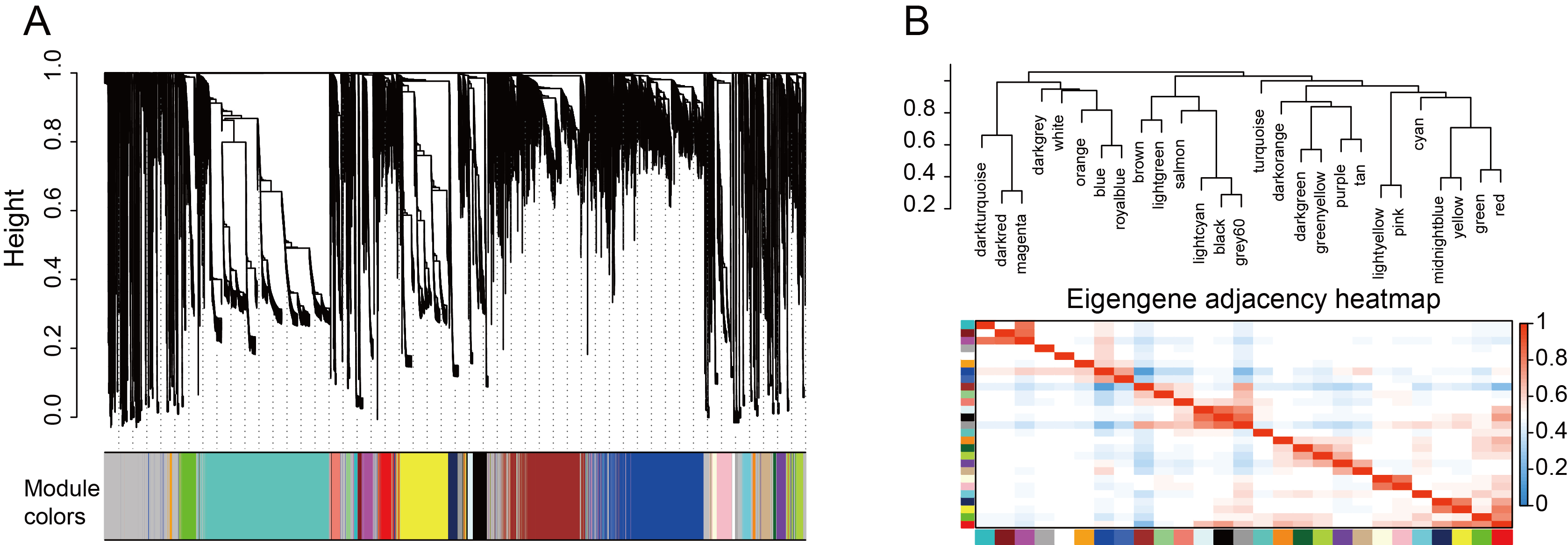

Supplement: Supplementary Figure 2 — (A) Network analysis dendrogram based on hierarchical clustering of genes by their topological overlap, identifies 27 modules. Colored bars below the dendrogram show module membership. (B) Heatmap of correlation between 27 co-expression modules. [file Image_2.JPEG]
